# Changes in the Concentration Profile of Selected Micro- and Macro-Elements in the Yellow Ligament Obtained from Patients with Degenerative Stenosis of the Lumbo-Sacral Spine

**DOI:** 10.3390/jcm14041252

**Published:** 2025-02-14

**Authors:** Damian Strojny, Dawid Sobański, Roman Wojdyła, Klaudia Skóra, Martyna Hoczela, Katarzyna Wyczarska-Dziki, Mateusz Miller, Mateusz Masternak, Rafał Staszkiewicz, Jerzy Wieczorek, Weronika Wieczorek-Olcha, Barbara Waltoś-Tutak, Paweł Gogol, Beniamin Oskar Grabarek

**Affiliations:** 1 Department of Neurology, New Medical Techniques Specjalist Hospital of St. Family in Rudna Mała, 36-060 Rzeszow, Poland; romanwojdyla@gmail.com (R.W.); kdziki@wsiz.edu.pl (K.W.-D.); 2 Collegium Medicum, WSB University, 41-300 Dąbrowa Górnicza, Poland; masternak.mat@gmail.com (M.M.); rafalstaszkiewicz830@gmail.com (R.S.); w.wieczorek.olcha@gmail.com (W.W.-O.); drpawelgogol@gmail.com (P.G.); bgrabarek7@gmail.com (B.O.G.); 3 Institute of Health Care, National Academy of Applied Sciences in Przemyśl, 37-700 Przemyśl, Poland; 4 Department of Neurosurgery, St. Raphael Hospital, 30-693 Krakow, Poland; 5 Department of Cardiology and Cardiovascular Interventions, University Hospital in Cracow, 30-668 Cracow, Poland; 6 Department of Neurological Rehabilitation, District Hospital of St. Padre Pio in Sędziszów Małopolski, 39-120 Sędziszów Małopolski, Poland; klaudiaskora26@gmail.com; 7 Nursing Faculty, Medical College, Universityof Information Technology and Managment in Rzeszow, 35-225 Rzeszow, Poland; mhoczela@wsiz.edu.pl; 8 Department of Neurology, Independent Public Healthcare Institution of the Ministry of Internal Affairs and Administration in Rzeszów, 35-111 Rzeszów, Poland; mateusz.d.miller@gmail.com; 9 Silesian Center for Rehabilitation and Manual Therapy ReVita in Mysłowice, 41-412 Mysłowice, Poland; 10 Department of Neurosurgery, 5th Military Clinical Hospital with the SP ZOZ Polyclinic in Krakow, 30-901 Krakow, Poland; 11 Department of Neurosurgery, Faculty of Medicine in Zabrze, Academy of Silesia in Katowice, 40-555 Katowice, Poland; 12 Department of Agricultural and Environmental Chemistry, University of Agriculture in Krakow, 31-120 Krakow, Poland; jerzy.wieczorek@urk.edu.pl; 13 Collegium Medicum, Uniwersytet Rzeszowski, 35-310 Rzeszow, Poland; barbara1waltos@gmail.com; 14 Department of Anesthesiology and Intensive Care, Our Lady of Perpetual Help Hospital in Wołomin, 05-200 Wołomin, Poland; 15 Department of Trauma and Orthopedic Surgery, Our Lady of Perpetual Help Hospital in Wołomin, 05-200 Wołomin, Poland; 16 Pain Treatment Clinic, Our Lady of Perpetual Help Hospital in Wołomin, 05-200 Wołomin, Poland

**Keywords:** ligamentum flavum hypertrophy, degenerative lumbo-sacral spinal stenosis, elemental analysis, pain intensity, inductively coupled plasma optical emission spectrometry (ICP-OES)

## Abstract

**Background/Objectives**: Degenerative lumbo-sacral spinal stenosis is characterized by spinal canal narrowing, often linked to ligamentum flavum hypertrophy. This study evaluated the elemental composition of ligamentum flavum tissue in DLSS patients compared to healthy controls. **Methods**: This study involved 180 patients diagnosed with degenerative lumbo-sacral spinal stenosis and 102 healthy controls. Ligamentum flavum samples were analyzed for concentrations of magnesium (Mg), calcium (Ca), phosphorus (P), zinc (Zn), copper (Cu), iron (Fe), sodium (Na), potassium (K), manganese (Mn), and lead (Pb) using inductively coupled plasma optical emission spectrometry (ICP-OES). Statistical analyses were conducted using Student’s *t*-test, ANOVA, and Pearson’s correlation, with a significance threshold of *p* < 0.05. **Results**: The study group exhibited significantly elevated levels of Mg (*p* < 0.001), Ca (*p* = 0.014), and P (*p* = 0.006), along with reduced concentrations of Zn (*p* = 0.021) and Cu (*p* = 0.038) compared to controls. No statistically significant differences were observed for Na, K, Mn, or Fe (*p* > 0.05). Elemental imbalances were more pronounced in individuals with higher body mass index (BMI) and varied by gender. Pain intensity demonstrated a significant correlation with Zn (*p* = 0.012) and Na (*p* = 0.045), but no consistent associations with Mg, Ca, or P. **Conclusions**: Altered Mg, Ca, P, and Zn levels in ligamentum flavum suggest their involvement in degenerative lumbo-sacral spinal stenosis pathophysiology. These elements may serve as potential biomarkers and therapeutic targets for mitigating spinal canal narrowing.

## 1. Introduction

Lumbar spinal stenosis is characterized by a reduction in the space of the spinal canal, leading to compression of neural and vascular elements within the lumbar region [1]. Among individuals in their 50s and 60s, acquired stenosis, also known as degenerative stenosis, is commonly observed. This condition is defined by a sagittal spinal canal dimension of less than 10–12 mm, which was initially normal in size [2,3]. The etiology of degenerative stenosis is multifactorial, with hypertrophic changes in the bony and ligamentous structures of the spinal canal playing a central role [4,5]. In addition to osteophyte formation and intervertebral disc degeneration, ligamentum flavum hypertrophy and calcification contribute significantly to spinal canal narrowing, exacerbating nerve root compression and clinical symptoms [6,7].

The clinical manifestations of degenerative lumbar spinal stenosis include pain in the lower extremities and buttocks, neurogenic claudication, and, less frequently, lower back pain. These symptoms worsen in upright positions or during walking but improve when sitting or bending forward [8,9,10]. Radiological imaging, including X-rays, computed tomography (CT), and magnetic resonance imaging (MRI), remains essential for diagnosis [11]. While conservative management is the first-line treatment, persistent symptoms often necessitate surgical decompression [12].

Trace elements and macronutrients play critical roles in maintaining the homeostasis and structural integrity of musculoskeletal tissues, including the ligamentum flavum. Macronutrients such as calcium (Ca), magnesium (Mg), and phosphorus (P) are integral to bone and soft tissue metabolism, while trace elements like zinc (Zn), copper (Cu), and manganese (Mn) regulate enzymatic activity, oxidative stress responses, and immune function [13]. Antioxidant trace elements such as Zn, Cu, and selenium (Se) help modulate inflammatory pathways and protect against oxidative damage, both of which contribute to degenerative spinal diseases. Zn, a component of Cu/Zn superoxide dismutase, has anti-inflammatory properties and promotes tissue repair, while Cu, as a cofactor for lysyl oxidase, is crucial for collagen and elastin cross-linking, supporting ligament strength. Deficiencies or imbalances in these elements may accelerate degenerative changes, predisposing individuals to ligamentum flavum hypertrophy and subsequent spinal stenosis [14,15,16].

Previous research on degenerative spine diseases has primarily focused on evaluating elemental concentrations in serum and intervertebral disc tissue to determine their role in tissue degeneration and pain severity [17,18,19,20,21]. Staszkiewicz et al. identified significant elemental alterations in degenerated intervertebral discs, suggesting that mineral imbalances contribute to disease progression, inflammatory responses, and extracellular matrix degradation [21]. Other studies have reported abnormal serum levels of Ca, Mg, Zn, and Cu in patients with degenerative spinal disorders, indicating systemic metabolic disturbances that may contribute to disease pathogenesis [14,22].

Despite these findings, limited research has investigated the elemental composition of ligamentum flavum tissue, a structure directly implicated in degenerative lumbar spinal stenosis. Unlike intervertebral discs, which primarily function as shock absorbers, the ligamentum flavum is subject to chronic mechanical stress, making it more susceptible to oxidative damage, mineral deposition, and inflammatory changes [23,24]. The ligament’s elastic fibers degrade and are gradually replaced by collagen, leading to fibrotic thickening, reduced elasticity, and hypertrophy—hallmarks of spinal canal narrowing [25]. Given the potential influence of micro- and macronutrients on collagen cross-linking, elastin degradation, and fibrotic remodeling, investigating their altered concentrations in ligamentum flavum tissue is essential for understanding disease progression and identifying potential therapeutic targets [26,27]. To address this knowledge gap, the present study aims to analyze the biochemical composition of ligamentum flavum tissue in patients with degenerative lumbar spinal stenosis, comparing elemental concentrations with non-degenerative tissue. By examining levels of iron (Fe), Zn, sodium (Na), potassium (K), Mg, P, and Ca, this study seeks to determine whether elemental imbalances contribute to ligament hypertrophy and fibrosis, potentially uncovering biomarkers for disease progression. A better understanding of these alterations may help develop therapeutic strategies aimed at restoring mineral homeostasis and mitigating ligament hypertrophy [28].

Considering the limited existing knowledge on the concentrations of micronutrients and macronutrients in patients with degenerative lumbo-sacral spinal stenosis, as well as the scarcity of research focused on this patient population, the objective of our study was to analyze the concentrations of Fe, Zn, Na, K, Mg, P, and Ca in the ligamentum flavum of individuals diagnosed with lumbo-sacral spinal degenerative stenosis, comparing these levels to those observed in non-degenerative tissue.

## 2. Materials and Methods

### 2.1. Study Design and Ethical Consideration

This is a prospective, case-control study designed to compare the elemental composition of ligamentum flavum tissue in patients diagnosed with degenerative lumbo-sacral spinal stenosis to healthy controls. A total of 180 patients with degenerative lumbo-sacral spinal stenosis and 102 healthy controls were included. All participants underwent thorough clinical and imaging evaluations to confirm their eligibility. The study was conducted in compliance with ethical guidelines, with approval from the Bioethics Committee of the State Academy of Applied Sciences in Przemyśl (approval number: 8/2024, on 1 August 2024). All biochemical analyses were performed under strictly controlled conditions, utilizing inductively coupled plasma optical emission spectrometry (ICP-OES) to accurately measure the concentrations of selected micro- and macroelements in ligamentum flavum tissue.

### 2.2. Study Group Characteristics

The study encompassed 180 individuals (87 women and 93 men) with a mean age of 51.12 ± 2.98 years, all diagnosed with degenerative stenosis of the lumbosacral spine (L/S). These patients were scheduled to undergo hemilaminectomy, with surgical eligibility determined and the procedures performed by a neurosurgery specialist (D.S.). A thorough neurological evaluation was conducted for each participant, consisting of both physical and subjective assessments, complemented by magnetic resonance imaging (MRI) using a scanner with a minimum strength of 1.5 Tesla (T). MRI protocols included SE T1, SE T1 fluid-attenuated inversion recovery, FSE T2, and short tau inversion recovery sequences, acquired in transverse and sagittal planes with slice thicknesses of 3 mm and 4 mm.

Anthropometric measurements, specifically body weight and height, were recorded to calculate the body mass index (BMI) for each participant.

Preoperative pain intensity was self-assessed by participants using a visual analog scale (VAS) ranging from 0 (no pain) to 10 (severe pain).

Participants confirmed that they had not followed any specific dietary regimen or consumed vitamin or mineral supplements classified as medicinal products in the six months preceding the study. Additionally, they reported no known instances of accidental exposure to heavy metal contaminants.

### 2.3. Control Group

The control group consisted of 102 healthy volunteers (49 women and 53 men) with an average age of 48.18 ± 1.87 years. These individuals exhibited no symptoms indicative of degenerative spinal stenosis or associated low back pain and had no history of osteoarthritis or other degenerative joint disorders. Additionally, none of the participants reported known accidental exposure to heavy metal contamination. As observed in the study group, control group participants reported no adherence to specific dietary regimens or use of medicinal vitamin or mineral supplements in the six months preceding the study.

### 2.4. Collection of Samples

The clinical material for the study was obtained from the 5th Military Clinical Hospital with the SP ZOZ Polyclinic in Krakow, St. Raphael Hospital, 30-693 Krakow, and the New Medical Techniques Specialist Hospital of the Holy Family in Rudna Mała.

The biochemical analysis was carried out at the Department of Agricultural and Environmental Chemistry, Faculty of Agricultural and Economic Sciences, Hugo Kołątaj University of Agriculture in Kraków. Element quantification was performed using inductively coupled plasma optical emission spectrometry (ICP-OES) to ensure precise measurements. For the analysis, ligamentum flavum samples from both the study and control groups were weighed, with dry masses ranging between 0.3 and 0.5 g. The samples underwent digestion in a mixture of concentrated suprapur-grade nitric acid (HNO_3_, 6 cm^3^) and hydrochloric acid (HCl, 1 cm^3^) supplied by Merck (Saint Louis, MO, USA). Digestion was conducted in a Multiwave 3000 microwave system (Anton Paar, Graz, Austria) using Teflon vessels. The process was executed at a maximum power of 1400 W for 25 min, consisting of a 10 min ramp-up phase to full power followed by 15 min of steady operation. After digestion, the samples were quantitatively filtered into 10 cm^3^ volumetric flasks using a 1% nitric acid solution. The concentrations of selected micro- and macronutrients were subsequently determined with the Optima 7300 Dual View atomic emission spectrometer (Perkin Elmer, Waltham, MA, USA), with each sample measured in triplicate to ensure data accuracy and reliability.

### 2.5. Statistical Analysis

Statistical analyses were conducted utilizing the Statplus software package (AnalystSoft Inc., Brandon, FL, USA, https://www.analystsoft.com/en/products/statplusmacle/). The significance level for all tests was established at (*p* < 0.05). As the initial dataset adhered to a normal distribution, verified through the Shapiro–Wilk test, parametric statistical methods were employed for the analysis.

Comparisons between two independent variables were performed using Student’s *t*-test, whereas differences among multiple variables were analyzed through one-way analysis of variance (ANOVA) with Tukey’s post hoc test for pairwise comparisons. Pearson’s correlation coefficient was calculated to explore relationships between the concentrations of elements, with a strong and statistically significant correlation being defined as a coefficient value of r > 0.60.

The relationship between lumbo-sacral stenosis and elemental concentrations was further assessed by calculating odds ratios (OR) based on 2 × 2 contingency tables. The interpretation of odds ratio values was as follows:

(OR} = 1): Indicates no difference in odds between the exposed and unexposed groups.
(OR} > 1): Suggests increased odds of disease occurrence in the exposed group.
(OR} < 1): Reflects reduced odds of disease occurrence in the exposed group.

#### Sample Size Analysis

The sample size was calculated using G*Power 3.1 software [29] based on effect size estimation from previous studies evaluating elemental composition in degenerative spinal diseases. A minimum sample size of 150 patients and 100 controls was determined to achieve a statistical power of 80% (β = 0.20) and an α-level of 0.05, ensuring sufficient sensitivity to detect significant differences in elemental concentrations. The actual study cohort of 180 patients and 102 controls exceeded this threshold, strengthening the reliability of the findings. Effect sizes (Cohen’s d) were computed for key elemental differences to assess their clinical relevance beyond statistical significance.

## 3. Results

### 3.1. Elemental Concentrations in Ligamentum Flavum: Study vs. Control Groups

The analysis of micro- and macronutrient concentrations in ligamentum flavum tissue revealed significant differences between the study and control groups (Table 1). Mg, Ca, P, and Pb levels were higher in the study group, with Mg showing the most pronounced difference (*p* < 0.001), followed by Ca (*p* = 0.014) and P (*p* = 0.006). In contrast, Zn and Cu concentrations were lower in the study group compared to controls, with Zn reaching statistical significance (*p* = 0.021) and Cu showing a trend toward significance (*p* = 0.086). No significant differences were observed for Fe, Mn, Na, or K (*p* > 0.05), suggesting that the alterations were specific to certain elements (Table 1).

### 3.2. Concentrations of the Selected Elements in the Study and Control Groups According to Gender

Gender-specific analysis indicated notable variations (Table 2), particularly in Cu and Pb levels. Women in the study group had lower Cu concentrations than controls (*p* = 0.054, borderline significance), while Pb concentrations were significantly higher in female participants (*p* = 0.032). Other elemental differences between men and women did not reach statistical significance (Table 2; *p* < 0.05).

### 3.3. Concentrations of Microelements and Macronutrients in the Study and Control Groups According to BMI Value

In the test group, 71 participants (37 women, 34 men) had a normal BMI (18.5–24.9), 63 participants (29 women, 34 men) were classified as overweight (BMI 25–29.9), and 46 participants (21 women, 25 men) were categorized as obese (BMI > 30).

In comparison, in the control group, 76 participants (37 women, 39 men) had a normal BMI, 19 participants (8 women, 11 men) were overweight, and 7 participants (4 women, 3 men) were obese, following the same BMI classification criteria.

Table 3 presents the concentrations of micro- and macronutrients in the serum samples from the study and control groups, categorized by BMI. Elemental concentrations were also analyzed according to BMI, revealing that Mg, Ca, and P levels were significantly higher in the study group across all BMI categories, with Ca (*p* = 0.012) and P (*p* = 0.021) showing particularly strong associations. Cu levels were lower in the study group across all BMI categories, with the most significant difference observed in individuals with normal BMI (*p* = 0.034).

### 3.4. Elemental Concentrations and Pain Intensity

None of the participants reported pain levels between 0 and 3. Pain severity distribution was as follows: 8 patients rated their pain at level 4, 14 at level 5, 17 at level 6, 9 at level 7, 4 at level 8, 4 at level 9, and 4 at level 10.

Table 4 presents the concentrations of selected elements in ligamentum flavum samples from the study group, categorized by pain intensity according to the VAS scale. Pain intensity analysis demonstrated a significant association between Zn concentrations and pain levels, with higher pain scores corresponding to lower Zn levels (*p* = 0.012). Na concentrations varied significantly with pain intensity (*p* = 0.045), suggesting a possible link between electrolyte balance and pain perception (Table 4).

### 3.5. Correlation Analysis of Elemental Concentrations

Correlation analysis within the study group revealed significant relationships between several elements, including positive associations between Zn and P (r = 0.62, *p* < 0.05) and between Mg and Ca (r = 0.74, *p* < 0.05), indicating potential metabolic interactions in ligamentum flavum tissue (Table 5).

In the control group (Table 6), several statistically significant relationships were observed, particularly between Na and K (r = 0.74), Zn and P (r = 0.73), and Ca and P (r = 0.95).

### 3.6. Risk Factor Analysis for Degenerative Lumbo-Sacral Spinal Stenosis

Table 7 summarizes the association between micro- and macronutrient concentrations and lumbo-sacral stenosis. The risk factor analysis demonstrated that subnormal levels of Fe, Zn, Na, Mg, K, P, and Ca were associated with significantly increased odds of degenerative lumbo-sacral spinal stenosis (*p* = 0.001 for all), with odds ratios ranging from 13.33 for Fe to 37.25 for Zn. Above-normal concentrations of these elements did not show a significant association with disease risk. These findings highlight the potential role of elemental imbalances in ligamentum flavum pathology and suggest the need for further investigations into their clinical implications.

## 4. Discussion

The rationale for this study is to elucidate the role of micro- and macronutrients in the pathophysiology of ligamentum flavum hypertrophy, a hallmark of degenerative lumbar spinal stenosis [18,30,31]. The balance of these elements is essential for enzymatic activity, oxidative stress modulation, and extracellular matrix homeostasis [17,32]. This study reveals significant alterations in elemental concentrations, particularly increased concentrations of Mg, P, and Ca, alongside reduced zinc concentrations in the study group, suggesting a potential role in disease progression.

The pronounced increase in Mg concentrations observed in ligamentum flavum tissue suggests its involvement in ligament hypertrophy through osteogenic and fibrotic pathways [33,34]. Mg is a cofactor in collagen synthesis, extracellular matrix remodeling, and calcium regulation, processes that directly influence ligament structure. While Mg is protective in joint degeneration [35], and has an analgesic effect in spinal osteoarthritis, its elevated levels in ligamentum flavum may indicate a dysregulated mineral metabolism contributing to hypertrophy.

Similarly, the increased P and Ca concentrations reinforce the hypothesis that aberrant mineralization contributes to ligamentum flavum hypertrophy [36,37]. These elements are critical for hydroxyapatite formation, essential for both bone and ligamentous structures [38]. Their role extends beyond structural support, as they participate in cellular signaling, osteogenesis, and extracellular matrix remodeling [38].

Excess Ca and P deposition in soft tissues can promote pathological calcification, reducing ligament elasticity and contributing to spinal canal narrowing. This process is similar to ectopic calcification in degenerative skeletal conditions [39,40].

Given that calcium–phosphorus metabolism is altered in systemic disorders such as osteoporosis and chronic kidney disease, these findings highlight the need for a broader metabolic approach to spinal stenosis pathophysiology [41].

The significantly reduced Zn concentrations in the study group suggest a compromised antioxidant defense system, potentially exacerbating oxidative damage, inflammation, and tissue fibrosis [42]. Zn is a critical regulator of oxidative stress, enzymatic activity, and extracellular matrix integrity, and its deficiency has been linked to degenerative musculoskeletal conditions, including osteoarthritis and osteoporosis. In ligamentum flavum tissue, Zn deficiency may promote fibrocartilaginous transformations and increased matrix degradation, contributing to hypertrophy [43,44,45,46]. Additionally, Zn deficiency is implicated in systemic disorders affecting multiple systems, including musculoskeletal, nervous, endocrine, and immune functions [42], further reinforcing its potential role in ligament degeneration.

Although elevated Pb levels in the study group were of borderline statistical significance, their potential contribution to ligamentum flavum hypertrophy should not be overlooked. Pb exposure has been linked to systemic inflammation, oxidative stress, and calcium metabolism disruption, all of which may contribute to degenerative changes [47,48]. While participants reported no known environmental lead exposure, the cumulative nature of Pb deposition over a lifetime raises concerns about its potential long-term impact on connective tissue integrity [49]. Previous research has identified Pb contamination in food sources [50,51], suggesting that dietary exposure may be an underestimated factor in ligamentum flavum degeneration.

Interestingly, our findings revealed no statistically significant differences in the concentrations of Na, K, Mn, and Fe between the study and control groups. This observation suggests that not all elements play an equally critical role in the pathogenesis of ligamentum flavum hypertrophy, although their physiological functions remain essential for overall homeostasis and health [52,53,54,55,56,57].

The influence of gender and BMI on elemental concentrations was evident in this study. Women in the study group exhibited lower Cu levels than controls (*p* = 0.054), while Mg levels were significantly elevated in both men and women (*p* < 0.001). These findings may be partially explained by hormonal differences, as estrogen regulates mineral metabolism and connective tissue remodeling [58,59,60]. Lower Cu levels in women may be linked to hormonal fluctuations, while the elevated magnesium concentrations across genders may indicate a broader dysregulation of mineral homeostasis in degenerative spinal conditions.

BMI-related differences in elemental concentrations suggest a potential link between obesity, chronic inflammation, and altered mineral metabolism [61,62]. Obese individuals exhibited the most significant deviations in Cu, Ca, Mg, and P levels, which aligns with systemic pro-inflammatory states associated with obesity [61,62,63,64]. Given that obesity accelerates oxidative stress and extracellular matrix remodeling, these findings suggest that BMI may influence ligament mineralization and hypertrophy. However, dietary intake, metabolic disorders, and systemic inflammation were not directly assessed, and future research should integrate nutritional assessments and metabolic profiling to clarify these associations.

Pain intensity analysis revealed a significant inverse correlation between Zn levels and pain severity (*p* = 0.012), suggesting that zinc depletion may exacerbate pain through increased oxidative stress and neuroinflammation [65,66]. Zn plays a role in glutamatergic neurotransmission, inflammatory signaling, and nociceptive processing, all of which are relevant to pain perception in degenerative spinal disorders [65,66]. Additionally, Na concentrations fluctuated with pain intensity (*p* = 0.045), potentially reflecting its role in neuronal excitability and pain modulation [65,66]. However, no clear trends were observed for Mg, Ca, and P concentrations in relation to pain, suggesting that these elements may primarily contribute to ligament hypertrophy rather than direct pain modulation.

The observed elemental alterations suggest potential applications as biomarkers for disease progression. Zn, Mg, and Ca levels could be used to identify individuals at higher risk of ligamentum flavum hypertrophy, enabling earlier diagnosis and intervention. Additionally, given their role in connective tissue integrity and inflammation, therapeutic strategies targeting mineral homeostasis—such as Zn supplementation or Mg modulation—could mitigate degenerative changes. However, this study does not establish clinical efficacy, and further research is needed to determine whether nutritional or pharmacological interventions can alter disease progression.

While this study provides valuable insights into the elemental composition of ligamentum flavum tissue in degenerative lumbo-sacral spinal stenosis, certain limitations should be considered. The cross-sectional design prevents establishment of causality, necessitating further longitudinal studies to clarify whether observed elemental imbalances contribute to or result from ligamentum flavum hypertrophy. Although the sample size was sufficient for statistical analysis, expanding the study to additional centers and diverse populations would enhance generalizability. The analysis relied solely on inductively coupled plasma optical emission spectrometry, a highly precise method for elemental quantification, but it lacked complementary histological, molecular, or imaging assessments that could further elucidate the biological and structural implications of these findings. Despite including a multicenter cohort, the exclusion criteria, particularly regarding hormonal and dietary influences, may have led to a non-representative sample, limiting broader applicability. While the study acknowledged the potential role of diet in mineral levels, it did not fully account for dietary variability, which could significantly impact elemental concentrations. Future research should incorporate detailed nutritional assessments to better evaluate the influence of diet. Additionally, although the study analyzed gender, body mass index, and pain intensity in relation to elemental concentrations, other potential confounders, including systemic inflammation, metabolic disorders, and environmental exposures, were not comprehensively assessed. Addressing these variables in future research would provide a more holistic understanding of the role of elemental imbalances in lumbar spinal stenosis pathology. Despite these considerations, this study provides a strong foundation for understanding biochemical alterations in ligamentum flavum tissue, offering valuable insights that could inform future research and potential therapeutic strategies for degenerative lumbar spinal stenosis.

## 5. Conclusions

This study provides new insights into the micro- and macronutrient composition of ligamentum flavum tissue in patients with lumbo-sacral spinal stenosis. The findings revealed significantly higher concentrations of Mg, Ca, P, and Pb in the study group compared to controls, while Zn and Cu levels were notably lower. Additionally, significant correlations were observed among elements such as Zn, P, and Ca, indicating potential metabolic alterations in ligament tissue. Elemental deficiencies, particularly in Zn, Mg, and Ca, were more prevalent in patients with lumbo-sacral spinal stenosis, suggesting a possible association with disease pathology. Variations in elemental concentrations were also influenced by BMI, gender, and pain intensity, emphasizing the need for further research to clarify these relationships. Future studies should focus on longitudinal analyses and mechanistic investigations to determine whether these changes contribute to ligamentum flavum hypertrophy or arise as a consequence of degenerative processes. Understanding these biochemical alterations may provide a basis for improved diagnostic approaches and potential therapeutic strategies for managing degenerative spinal conditions.

## Figures and Tables

**Table 1:** Concentrations of the selected elements in the ligamentum flavum samples of the study and control groups.

Micro-/Macroelements	Study Group Concentration [mg/kg d.m.]	Control Group Concentration [mg/kg d.m.]	*p* (*t*-Student’s Test)
Cu	2.43 ± 1.22 (1.68–3.18)	3.77 ± 1.89 (2.60–4.94)	0.086
Fe	145.47 ± 57.73 (109.69–181.25)	124.11 ± 51.38 (92.26–155.96)	0.296
Mn	0.45 ± 0.23 (0.31–0.59)	0.44 ± 0.22 (0.30–0.58)	0.446
Pb	0.97 ± 0.48 (0.67–1.27)	0.59 ± 0.29 (0.41–0.77)	0.067
Zn	32.78 ± 16.39 (22.62–42.94)	22.23 ± 11.12 (15.34–29.12)	0.021
Na	14,536.27 ± 7268.14 (10,031.43–19,041.11)	13,492.97 ± 1962.21 (12,276.78–14,709.16)	0.689
Mg	55,756.86 ± 27,878.43 (38,477.63–73,036.09)	126.22 ± 63.11 (87.10–165.34)	<0.001
K	313.15 ± 156.57 (216.10–410.20)	305.80 ± 145.13 (215.85–395.75)	0.628
Ca	5463.94 ± 2731.97 (3770.65–7157.23)	1532.36 ± 722.63 (1084.47–1980.25)	0.014
P	5301.50 ± 2650.75 (3658.55–6944.45)	1425.29 ± 712.64 (983.59–1866.99)	0.006

Mean ± standard deviation (95% Cl, 95% Confidence intervals); Fe, iron; Zn, zinc; Na, sodium; Mg, magnesium; K, potassium; P, phosphorus; and Ca, calcium.

**Table 2:** Concentrations of the selected elements in women and men in the study and control groups.

Micro-/Macroelements	Gender	Study Group Concentration [mg/kg d.m.]	Control Group Concentration [mg/kg d.m.]	*p* (*t*-Student’s Test)
Cu	Women	2.31 ± 1.16 (1.59–3.03)	3.58 ± 1.79 (2.47–4.69)	0.054
	Men	2.55 ± 1.27 (1.76–3.34)	3.96 ± 1.98 (2.73–5.19)	
Fe	Women	138.20 ± 49.35 (107.61–168.79)	117.90 ± 51.38 (86.05–149.75)	0.313
	Men	152.74 ± 63.06 (113.66–191.82)	130.32 ± 63.06 (91.24–169.40)	
Mn	Women	0.43 ± 0.21 (0.30–0.56)	0.42 ± 0.21 (0.29–0.55)	0.410
	Men	0.47 ± 0.23 (0.32–0.62)	0.46 ± 0.23 (0.32–0.60)	
Pb	Women	0.92 ± 0.46 (0.63–1.21)	0.56 ± 0.28 (0.39–0.73)	0.032
	Men	1.02 ± 0.51 (0.70–1.34)	0.62 ± 0.31 (0.43–0.81)	
Zn	Women	31.14 ± 15.57 (21.49–40.79)	21.12 ± 10.56 (14.57–27.67)	0.081
	Men	34.42 ± 17.21 (23.75–45.09)	23.34 ± 11.67 (16.11–30.57)	
Na	Women	13,809.46 ± 6904.73 (9529.86–18,089.06)	12,818.32 ± 1962.21 (11,602.13–14,034.51)	0.511
	Men	15,263.08 ± 7631.54 (10,533.00–19,993.16)	14,167.62 ± 7083.81 (9777.03–18,558.21)	
Mg	Women	52,969.02 ± 26,484.51 (36,553.75–69,384.29)	119.91 ± 59.95 (82.75–157.07)	<0.001
	Men	58,544.70 ± 29,272.35 (40,401.51–76,687.89)	132.53 ± 66.27 (91.46–173.60)	
K	Women	297.49 ± 148.75 (205.30–389.68)	290.51 ± 145.13 (200.56–380.46)	0.081
	Men	328.81 ± 164.41 (226.91–430.71)	321.09 ± 160.54 (221.58–420.60)	
Ca	Women	5190.74 ± 2595.37 (3582.11–6799.37)	1455.74 ± 722.63 (1007.85–1903.63)	0.010
	Men	5737.14 ± 2868.57 (3959.18–7515.10)	1608.98 ± 804.49 (1110.35–2107.61)	
P	Women	5036.43 ± 515.50 (4716.92–5355.94)	1354.03 ± 677.01 (934.41–1773.65)	0.004
	Men	5566.57 ± 434.98 (5296.97–5836.17)	1496.55 ± 434.98 (1226.95–1766.15)	

Mean ± standard deviation (95% Cl, 95% Confidence intervals); Fe, iron; Zn, zinc; Na, sodium; Mg, magnesium; K, potassium; P, phosphorus; and Ca, calcium.

**Table 3:** Concentrations of microelements and macronutrients in the study and control groups according to BMI value.

Micro-/Macroelements	BMI Category	Study Group Concentration [mg/kg d.m.]	Control Group Concentration [mg/kg d.m.]	*p* (*t*-Student’s Test)
Cu	Normal	2.41 ± 1.21 (1.66–3.16)	3.73 ± 1.86 (2.57–4.89)	0.034
	Overweight	2.48 ± 1.24 (1.71–3.25)	3.84 ± 1.92 (2.65–5.03)	
	Obese	2.78 ± 1.39 (1.92–3.64)	4.32 ± 2.16 (2.98–5.66)	
Fe	Normal	142.86 ± 49.35 (112.27–173.45)	121.88 ± 51.38 (90.03–153.73)	0.388
	Overweight	147.62 ± 49.25 (117.09–178.15)	125.94 ± 51.58 (93.97–157.91)	
	Obese	158.26 ± 49.05 (127.86–188.66)	135.02 ± 51.68 (102.99–167.05)	
Mn	Normal	0.45 ± 0.23 (0.31–0.59)	0.44 ± 0.22 (0.30–0.58)	0.551
	Overweight	0.45 ± 0.23 (0.31–0.59)	0.44 ± 0.22 (0.30–0.58)	
	Obese	0.50 ± 0.25 (0.35–0.65)	0.49 ± 0.24 (0.34–0.64)	
Pb	Normal	1.01 ± 0.51 (0.70–1.32)	0.61 ± 0.30 (0.42–0.80)	0.494
	Overweight	1.01 ± 0.51 (0.70–1.32)	0.62 ± 0.31 (0.43–0.81)	
	Obese	1.08 ± 0.54 (0.75–1.41)	0.66 ± 0.30 (0.47–0.85)	
Zn	Normal	33.13 ± 16.57 (22.86–43.40)	22.47 ± 11.23 (15.51–29.43)	0.512
	Overweight	34.59 ± 17.30 (23.87–45.31)	23.46 ± 11.73 (16.19–30.73)	
	Obese	37.53 ± 18.77 (25.90–49.16)	25.45 ± 12.72 (17.56–33.34)	
Na	Normal	14,714.63 ± 7357.31 (10,154.52–19,274.74)	13,658.53 ± 1962.21 (12,442.34–14,874.72)	0.712
	Overweight	15,798.78 ± 7899.39 (10,902.69–20,694.87)	14,664.87 ± 1962.21 (13,448.68–15,881.06)	
	Obese	15,810.37 ± 7905.19 (10,910.69–20,710.05)	14,675.62 ± 1962.21 (13,459.43–15,891.81)	
Mg	Normal	57,844.52 ± 28,922.26 (39,918.32–75,770.72)	130.95 ± 65.47 (90.37–171.53)	<0.001
	Overweight	58,036.66 ± 29,018.33 (40,050.91–76,022.41)	131.38 ± 65.69 (90.66–172.10)	
	Obese	63,993.44 ± 31,996.72 (44,161.67–83,825.21)	144.87 ± 72.44 (99.97–189.77)	
K	Normal	311.72 ± 155.86 (215.12–408.32)	304.41 ± 145.13 (214.46–394.36)	0.612
	Overweight	341.70 ± 170.85 (235.81–447.59)	333.68 ± 142.13 (245.59–421.77)	
	Obese	349.16 ± 174.58 (240.95–457.37)	340.97 ± 147.41 (249.60–432.34)	
Ca	Normal	5676.78 ± 2838.39 (3917.53–7436.03)	1592.05 ± 722.63 (1144.16–2039.94)	0.012
	Overweight	5931.62 ± 2965.81 (4093.39–7769.85)	1663.52 ± 728.32 (1212.10–2114.94)	
	Obese	5811.28 ± 2905.64 (4010.35–7612.21)	1629.77 ± 711.23 (1188.95–2070.59)	
P	Normal	5102.98 ± 515.50 (4783.47–5422.49)	1371.92 ± 685.96 (946.76–1797.08)	0.021
	Overweight	5358.55 ± 515.50 (5039.04–5678.06)	1440.63 ± 720.32 (994.17–1887.09)	
	Obese	5829.82 ± 515.50 (5510.31–6149.33)	1567.33 ± 783.66 (1081.61–2053.05)	

Mean ± standard deviation (95% Cl, 95% Confidence intervals); Fe, iron; Zn, zinc; Na, sodium; Mg, magnesium; K, potassium; P, phosphorus; and Ca, calcium.

**Table 4:** Concentrations of the selected elements in the ligamentum flavum from the study group depending on pain intensity.

Micro-/ Macroelements	Pain Intensivity According to VAS							*p*-Value (ANOVA)
	4	5	6	7	8	9	10	
Cu	3.67 ± 1.83 (2.53–4.81)	3.73 ± 1.86 (2.57–4.89)	1.24 ± 0.62 (0.86–1.62)	2.00 ± 1.00 (1.38–2.62)	2.31 ± 1.16 (1.59–3.03)	2.69 ± 1.34 (1.86–3.52)	0.87 ± 0.33 (0.67–1.07)	0.178
Fe	159.31 ± 72.40 (114.44–204.18)	143.87 ± 52.23 (111.50–176.24)	144.58 ± 51.99 (112.36–176.80)	164.38 ± 71.55 (120.03–208.73)	149.49 ± 63.11 (110.37–188.61)	138.30 ± 59.31 (101.54–175.06)	129.98 ± 49.05 (99.58–160.38)	0.345
Mn	0.43 ± 0.21 (0.30–0.56)	0.54 ± 0.27 (0.37–0.71)	0.42 ± 0.21 (0.29–0.55)	0.97 ± 0.48 (0.67–1.27)	0.33 ± 0.17 (0.23–0.43)	0.15 ± 0.07 (0.10–0.20)	0.20 ± 0.10 (0.14–0.26)	0.067
Pb	0.99 ± 0.49 (0.68–1.30)	1.04 ± 0.52 (0.72–1.36)	1.05 ± 0.53 (0.72–1.38)	0.72 ± 0.36 (0.50–0.94)	0.98 ± 0.49 (0.68–1.28)	1.75 ± 0.88 (1.21–2.29)	0.89 ± 0.45 (0.61–1.17)	0.285
Zn	34.53 ± 17.27 (23.83–45.23)	49.17 ± 24.59 (33.93–64.41)	29.59 ± 14.79 (20.42–38.76)	33.51 ± 15.77 (23.74–43.28)	31.76 ± 12.93 (23.75–39.77)	20.03 ± 10.02 (13.82–26.24)	20.38 ± 10.19 (14.06–26.70)	0.012
Na	14,283.33 ± 7141.66 (9856.88–18,709.78)	11,949.09 ± 5974.55 (8246.03–15,652.15)	11,573.47 ± 5786.73 (7986.81–15,160.13)	17,103.67 ± 8551.83 (11,803.19–22,404.15)	23,110.00 ± 11,555.00 (15,948.14–30,271.86)	26,733.33 ± 13,366.67 (18,448.59–35,018.07)	8700.00 ± 3335.67 (6632.53–10,767.47)	0.045
Mg	42,560.79 ± 21,280.40 (29,371.06–55,750.52)	38,477.23 ± 19,238.62 (26,553.01–50,401.45)	104,701.59 ± 52,350.79 (72,254.23–137,148.95)	68,470.70 ± 34,235.35 (47,251.41–89,689.99)	149,490.00 ± 63,112.89 (110,372.23–188,607.77)	138,300.00 ± 59,309.11 (101,539.83–175,060.17)	129,975.00 ± 49,046.74 (99,575.52–160,374.48)	0.501
K	208.50 ± 95.19 (149.50–267.50)	317.56 ± 158.78 (219.15–415.97)	367.18 ± 183.59 (253.39–480.97)	630.55 ± 176.19 (521.35–739.75)	326.00 ± 163.00 (224.97–427.03)	150.00 ± 20.00 (137.60–162.40)	155.00 ± 25.00 (139.50–170.50)	0.223
Ca	8591.87 ± 4295.94 (5929.22–11,254.52)	9804.29 ± 4902.15 (6765.91–12,842.67)	3358.17 ± 1679.09 (2317.46–4398.88)	13,544.17 ± 3917.26 (11,116.23–15,972.11)	982.00 ± 491.00 (677.68–1286.32)	1753.33 ± 876.66 (1209.97–2296.69)	887.50 ± 443.75 (612.46–1162.54)	0.429
P	5087.33 ± 2543.66 (3510.75–6663.91)	6754.10 ± 3377.05 (4660.98–8847.22)	3669.00 ± 1834.50 (2531.97–4806.03)	7663.17 ± 3831.59 (5288.33–10,038.01)	3176.10 ± 1292.80 (2374.81–3977.39)	2903.00 ± 1114.24 (2212.39–3593.61)	2038.00 ± 1019.00 (1406.42–2669.58)	0.309

Mean ± standard deviation (95% Cl, 95% Confidence intervals); Fe, iron; Zn, zinc; Na, sodium; Mg, magnesium; K, potassium; P, phosphorus; Ca, calcium; and VAS, visual analog scale.

**Table 5:** Correlations between the individual concentrations of the elements in the serum samples from the study group.

Microelement/Macronutrient	Cu	Fe	Zn	Na	Mg	K	P	Ca
Cu	1.00	0.12	0.11	−0.21	0.41 *	0.21	−0.31	−0.12
Fe	0.12	1.00	0.38 *	0.33 *	0.21	0.26	0.48 *	0.22
Zn	0.11	0.38 *	1.00	−0.41	−0.16	0.51 *	0.62 *	0.89 *
Na	−0.21	0.33 *	−0.41	1.00	0.31	0.62 *	−0.06	0.15
Mg	0.41 *	0.21	−0.16	0.31	1.00	−0.10	0.15	0.74 *
K	0.21	0.26	0.51 *	0.62 *	−0.10	1.00	0.33	0.32
P	−0.31	0.48 *	0.62 *	−0.06	0.15	0.33	1.00	0.92 *
Ca	−0.12	0.22	0.89 *	0.15	0.74 *	0.32	0.92 *	1.00

* Statistically significant correlation; Fe, iron; Zn, zinc; Na, sodium; Mg, magnesium; K, potassium; P, phosphorus; and Ca, calcium.

**Table 6:** Correlations between the individual concentrations of the elements in the serum samples from the control group.

Microelement/Macronutrient	Cu	Fe	Zn	Na	Mg	K	P	Ca
Cu	1.00	0.21	−0.32	0.13	0.03	−0.18	0.12	0.25
Fe	0.21	1.00	0.29	−0.18	−0.59	−0.46	0.33	−0.09
Zn	−0.32	0.29	1.00	0.26	0.22	0.47	0.73 *	−0.07
Na	0.13	−0.18	0.26	1.00	0.49	0.74 *	0.25	0.39
Mg	0.03	−0.59	0.22	0.49	1.00	0.15	0.36	0.52
K	−0.18	−0.46	0.47	0.74 *	0.15	1.00	0.28	0.11
P	0.12	0.33	0.73 *	0.25	0.36	0.28	1.00	0.95 *
Ca	0.25	−0.09	−0.07	0.39	0.52	0.11	0.95 *	1.00

* Statistically significant correlation; Fe, iron; Zn, zinc; Na, sodium; Mg, magnesium; K, potassium; P, phosphorus; and Ca, calcium.

**Table 7:** Association of L/S stenosis with concentration of microelements and macronutrients in ligamentum flavum samples of the study group.

Microelement/Macronutrient	Exposure	Study Group	Control Group	Odds Ratio	95% CI	*p*-Value
Cu	In the norm	98	69	Referent		
	Under the norm	41	30	2.19	2.01; 3.54	0.045
	Above the norm	41	3	0.11	0.03; 0.81	0.76
Fe concentration	In the norm	70	56	Referent		
	Under the norm	60	3	13.33	4.52; 39.31	0.001
	Above the norm	50	43	0.95	0.48; 1.90	0.89
Zn concentration	In the norm	65	51	Referent		
	Under the norm	75	3	37.25	8.13; 170.66	0.001
	Above the norm	40	48	1.96	0.39; 9.95	0.39
Na concentration	In the norm	68	54	Referent		
	Under the norm	62	3	31.00	7.05; 136.35	0.001
	Above the norm	50	45	1.04	0.50; 2.15	0.92
Mg concentration	In the norm	64	49	Referent		
	Under the norm	72	4	18.00	4.87; 66.51	0.001
	Above the norm	44	49	0.91	0.42; 1.97	0.82
K concentration	In the norm	66	53	Referent		
	Under the norm	70	2	28.00	6.45; 121.55	0.001
	Above the norm	44	47	0.89	0.38; 2.06	0.79
P concentration	In the norm	63	54	Referent		
	Under the norm	65	3	27.86	6.17; 125.76	0.001
	Above the norm	52	45	0.95	0.41; 2.20	0.91
Ca concentration	In the norm	65	51	Referent		
	Under the norm	68	3	33.33	7.86; 141.45	0.001
	Above the norm	47	48	0.94	0.38; 2.32	0.89

Data are presented as number of cases; 95% Cl, 95% Confidence intervals; Fe, iron; Zn, zinc; Na, sodium; Mg, magnesium; K, potassium; P, phosphorus; and Ca, calcium.

## Data Availability

All data generated or analyzed during this study are included in this published article.
